# Extraction of the ^131^Cs from neutron irradiated barium oxide under microwave radiation

**DOI:** 10.1007/s10967-013-2687-4

**Published:** 2013-08-21

**Authors:** S. Khujaev, A. Vasidov, E. A. Markelova

**Affiliations:** Institute of Nuclear Physics, Tashkent, Uzbekistan

**Keywords:** Extraction of ^131^Cs, Microwave radiation, Neutron irradiation, Brachytherapy

## Abstract

In this work, the solubility dependence of radioactive baric samples under influence of microwave radiation (MWR) has been investigated at various concentrations of HCl after irradiation of BaO target at a flux of thermal neutrons. It is established that the use of MWR for dissolution of a sparingly-soluble barium oxide will accelerate a dissolution process at almost an order of magnitude in comparison with usual heating. Each time after separation of the ^131^Cs from radioactive barium solution analyses on radionuclide purity of the ^131^Cs product solutions were carried out.

## Introduction

The appearance of ^131^Cs implants at the radiopharmaceutical market and its clinical use in brachytherapy has made a breakthrough in prostate cancer treatment. The repopulation rate of the prostate cancer cells is 1.5 % a day, and it doubles in 67 days. The absorption of 90 % of radiation doze by cancer cells will require three half-life periods of radionuclides. Therefore, the destruction rate of cancer cells by ^131^Cs (*T*_1/2_ = 9.6 days) is essentially higher than repopulation rate, and practically does not give a chance for their survival, as opposed to ^103^Pd (17 days) and ^125^I (59.9 days) [1, 2].

To obtain ^131^Cs, the most useful nuclear reaction with thermal neutrons is [3]:

1 $$ ^{130}{\text{Ba }}(n,{{\upgamma}})^{131}{\text{Ba}} \to^{131}{\text{Cs}} $$

Parent radionuclide ^131^Ba with *T*_1/2_ = 11.8 days decays to daughter radionuclide ^131^Cs which decays to stable ^131^Xe.

In Table 1, nuclear-physical characteristics and radioactivity values of radionuclides at the irradiation of 1 gram of natural barium by a thermal neutron flux of 3.5 × 10^13^ cm^−2^ s^−1^ for 5 days on (*n*, γ) reaction are shown [7, 8].

**Table 1:** Nuclear-physical characteristics of the Ba radionuclides produced by (*n*, γ) reaction [7, 8]

Isotope (abundance, %)	Radionuclide	*T*_1/2_	Cross section, barn	Activity, Bq
^130^Ba (0.106)	^131^Ba	11.8 days	10	4.38 × 10^8^
	^131m^Ba	14.6 min	2.5	4.1 × 10^8^
^132^Ba (0.097)	^133^Ba	10.5 year	7	9.7 × 10^5^
^134^Ba (2.42)	^135m^Ba	1.2 days	0.16	5.7 × 10^8^
^136^Ba (7.85)	^137m^Ba	2.55 min	0.011	1.34 × 10^8^
^138^Ba (71.7)	^139^Ba	1.38 h	0.36	3.94 × 10^10^
^131^Cs	^132^Cs	6.47 days	250 [7]	5.5 × 10^3^

As shown in Table 1, radioactivity yield is only 4.38 × 10^8^ Bq ^131^Ba, since the nuclear reaction is limited by the low-abundance isotope of the ^130^Ba (0.1 %). The discharge of ^131^Cs essentially leads to the separation of some tens of nanograms of ^131^Cs from very high amount of highly-active barium radionuclides [4, 5].

In scientific literature and patents, some radiochemical methods of extraction of ^131^Cs from irradiated BaCO_3_, Ba(NO_3_)_2_ are described [3–7]. And also there are the studies of mutual extraction of cesium and barium radionuclides from the radioactive wastes by calcium alginate beads [9].

In our case, barium oxide has been taken as a target material, since a percent share of natural barium in compounds consists of 89.5 % in BaO, 52 % in Ba(NO_3_)_2_, 69 % in BaCO_3_ and 65 % in BaCl_2_·2H_2_O. Therefore, irradiation of BaO yields a higher radioactivity of ^131^Ba than irradiation with identical mass of Ba compounds.

The reason why barium oxide has not been used as a target material is the long irradiation of large amounts of BaO at the nuclear reactor for 5–7 days which leads to some structural changes. As a result, the further process of solution becomes very difficult at heating and takes a long time in acid medium.

From this point of view, the process of dissolution of a sparingly-soluble radioactive samples under influence of microwave radiation represents scientific and practical interest.

The microwave chemistry was formed in 1980 at a joint of physics and chemistry. It studies chemical processes in solid and liquid materials, connected with the use of energy of the microwave radiation [10]. Therefore, different compounds convert microwave radiation to heat by different amounts.

The aim of this work was to study the solubility dependence of irradiated barium oxide on various concentration of a hydrochloric acid under influence of microwave radiation.

## Experimental

As a source of MWR, a normal domestic MWR oven was used, with following technical parameters: model—NIKAI® NMO-517; voltage of 220-240 Volt at 50 Hz; MWR power of 120–700 W at operating frequency 2.450 GHz.

For dissolution of irradiated samples in a microwave oven, the special vessel and auxiliary accessories were made of Teflon material (a cup, a lid, a mixer and a holder). It is known that at the certain frequency and temperature the dielectric material and solution have different capacities of absorption of microwave radiation and various abilities to convert it in thermal energy, which is characterized by the concept of a dissipation coefficient. At frequency 2.450 GHz and temperature 25 °C, the value of dissipation coefficient varies 1,000 times depending of the compound material. For example, dissipation coefficient is equal to 157 for water, and it is ≪1 for Teflon [10].

**Fig. 1:**
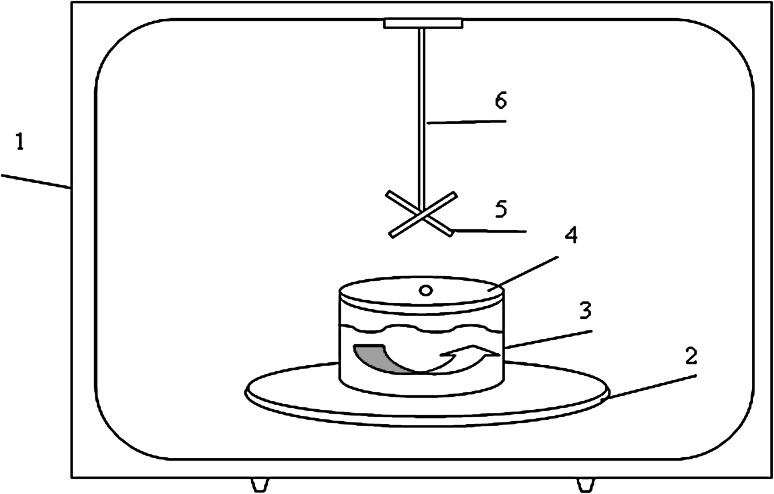
Schematic view of MWR oven and Teflon accessories for dissolution of irradiated samples. *1* microwave oven, *2* rotary quarts plate, *3* cup for radioactive solution, *4* lid, *5* mixer, *6* mixer holder

In Fig. 1, the schematic view of experimental MWR oven for dissolution of the irradiated samples is shown.

As shown in Fig. 1, in MWR oven (1) on the center of a quartz plate (2) a cup with a solution of irradiated BaO in a hydrochloric acid (3) is fixed. The mixer (5) is installed into the solution and closed by a lid (4), the end of mixer is fastened to the motionless holder (6). The mixing process of baric solution is carried out by rotating the cup with quartz plate, while mixer is fixed.

The studied barium oxide (*m*_Ba_ = 20 g) is sealed in quartz vessel and is located in aluminum block-containers. It is irradiated at a thermal neutron flux of 5 × 10^13^ cm^−2^ s^−1^ for 5 days, at the WWR SM reactor of INP AS RUz. The irradiated samples of BaO were aged for 10 days for the maximal accumulation of radioactivity of ^131^Cs and decay of radionuclides with *T*_1/2_ < 1 days.

## Results and discussions

**Fig. 2:**
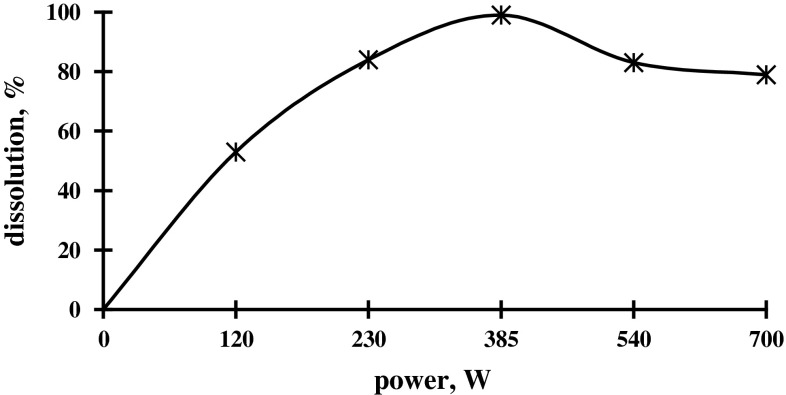
Dependence of dissolution of irradiated BaO on power of MWR

To determine optimum power of MWR and necessary time for a full dissolution of the radioactive BaO, the sample of BaO, weighing 3 g, in stehiometric quantity (14 mL) 1 M HCl was taken, and its dissolution at five various power levels of MWR was carried out. In Fig. 2, the dependence of dissolution of irradiated BaO on the power of MWR oven was shown. At 120 and 230 W, 50 and 75 % dissolution of BaO in 1 M HCl were observed, respectively. The full dissolution of BaO was occurred at 385 W in 150 s, and the further increase in MWR power did not give desirable effect. The growth of power of MWR leads to change of color solution.

At 540 W, the solution gets white-yellow coloration and then, at 700 W, becomes dark yellow. As it is shown in Fig. 2, for full dissolution of irradiated BaO, an optimal power of MWR is 385 W.

**Fig. 3:**
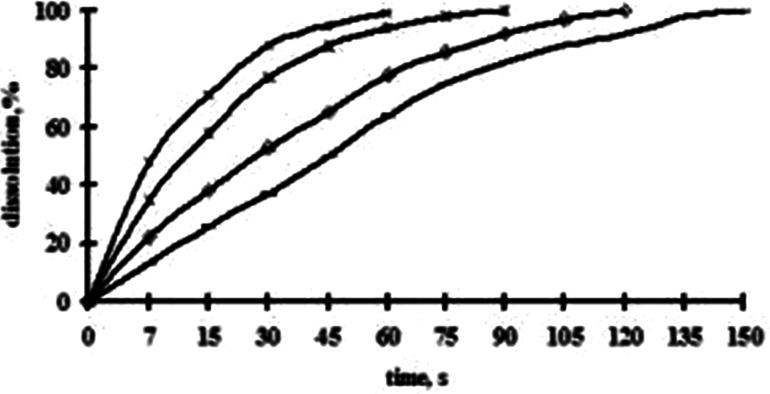
Dependence of dissolution time of irradiated BaO on concentrations of HCl

The study of influence of the hydrochloric acid concentration on solubility of irradiated BaO showed that the increase of HCl concentration from 1 to 5 M reduces the dissolution time from 150 to 60 s (Fig. 3). However, dissolution of BaO in strongly concentrated mediums is undesirable, since the release of radioactive solution from the cups to the MWR oven occurs.

**Fig. 4:**
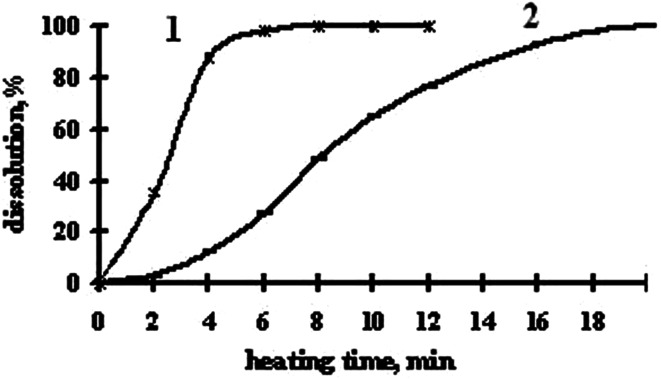
Dependence of dissolution of an irradiated BaO on heating time for: *1* microwave and *2* electric ovens

The times of the full dissolution of the radioactive samples were established, as at the usual and MWR heating, the results are shown in Fig. 4.

It is established that at usual heating on the electric oven the full dissolution of BaO occurs in ≥20 min, and at MWR heating it occurs in ≤2.5 min. Besides, at dissolution under the influence of MWR, strictly a stoichiometric volume of hydrochloric acid is used, which leads to the decrease of the volumes of highly radioactive solutions and of the reprocessing time of irradiated samples. Therefore, the technological process of obtain ^131^Cs is simplified as well.

After a full dissolution of the BaO, ^131^Cs is extracted by adding of 1 M Na_2_CO_3_ solution to sedimentation ions of Ba^2+^, as a carbonate form, in the alkalescent medium according to reaction:

2 $$ {\text{BaCl}}_{ 2} + {\text{Na}}_{ 2} {\text{CO}}_{ 3} \to {\text{BaCO}}_{ 3} + {\text{ 2NaCl}} $$

The obtained solution of ^131^Cs was extracted from the precipitate of BaCO_3_ by filtration and was rinsed with 0.1 M ammonium hydrate solution. The traces of barium ions in the filtrate were removed with the addition of 3–5 mL 5 M H_2_SO_4_ solution for precipitation in barium sulfate form. Because the ^131^Cs (*T*_1/2_ = 9.6 days) is daughter radionuclide of the ^131^Ba (*T*_1/2_ = 11.8 days), the equilibrium radioactivity between ^131^Ba and ^131^Cs is established in 10–15 days. In our experiments, four cycles of branching of ^131^Cs from ^131^Ba or the “milking” process were held every 10 days by dissolution of the precipitate of BaCO_3_ in hydrochloric acid each time.

### Radionuclidic purity

The radionuclidic purity of ^131^Cs product and its radiochemical yield were investigated by gamma-ray spectrometry using HPGe and Si(Li) detectors from CANBERRA (USA) after each cycle of separation of the ^131^Cs from barium solution. When analyzing at the X-ray Spectrometer (Si(Li) detector), only two lines of Cs → Xe transitions 29.67 and 33.61 keV were recognized. The measurements of the background and solution radioactivity were carried out on HPGe detector in 600–4000 s.

The activity value of the radionuclide was determined by the formula:

3 $$ A \, = \, s \cdot k_{1} \cdot k_{2} /t_{\text{ms}} \cdot \alpha_{\text{eff}} \cdot \eta_{\gamma } $$

where *A* radionuclide activity, Bq; *s* peak area, imp; *k*_1_ correction on geometry of measurements; *k*_2_ correction on aliquot; *t*_ms_ measuring time activity, *s*; *α*_eff_ registration efficiency of detectors for gamma and X-ray radiations, relative unit; *η*_γ_ intensity of gamma and X-ray radiations, relative unit. The root-mean-square error of this method is no more than ± 10 %.

In Table 2, the results of radionuclide activity measurements are presented on four cycles of separation of the ^131^Cs at a rate per 1 g of barium.

**Table 2:** The radionuclidic purity of the ^131^Cs solutions

Target	Radio-nuclide	I-cycle	II-cycle	III-cycle	IV-cycle
		A, Bq	A, Bq	A, Bq	A, Bq
BaO	^131^Ba	940	1,460	810	357
	^131^Cs	1.4 × 10^7^	3.9 × 10^7^	1.2 × 10^7^	3.6 × 10^6^
	^132^Cs	1,690	–	–	–
	^124^Sb	3,980	9,000	770	190

As shown in Table 2, the radionuclide ^124^Sb is presented in all cycles of separations. Apparently, it may be explained by the presence of natural antimony in basic samples of BaO. Its contents in cesium solutions were determined by relative method in an interval 2.5 × 10^−8^–8.7 × 10^−7^ g/g. The percent ratio of radioactivity of the ^124^Sb/^131^Cs in the 1th–4th cycles of extractions was 0.028, 0.023, 0.0064 and 0.0052 %, respectively. In all cesium solutions, ^132^Cs has been found only at the first cycle of extraction and its contribution to the ^131^Cs activity accounted to 0.012 %. The contribution of the parent radionuclide ^131^Ba to the ^131^Cs solutions was on the average 0.0067 %.

## Conclusion

Thus, the use of the MWR accelerates the dissolution process of the radioactive sample, almost an order of magnitude compared to the heat of electric oven, and takes ≤2.5 min at 385 W and at concentration 1 M HCl, which considerably simplifies technological process. The increase of solution concentrations from 1 to 5 M HCl also leads to the reduction of dissolution time from 150 to 60 s of irradiated BaO.

Radiochemical purity of the ^131^Cs product was determined to be about 99.97 %. The percentage relations of impurity activities to ^131^Cs product were ^124^Sb/^131^Cs −0.015 %; ^132^Cs/^131^Cs −0.012 % and ^131^Ba/^131^Cs−0.0067 %.

This work was supported by A11-FA-F131 Grant of Committee for coordination of science and technology development under the Cabinet of Ministers of Uzbekistan.
